# 盐酸埃克替尼治疗晚期复发非小细胞肺癌的临床疗效

**DOI:** 10.3779/j.issn.1009-3419.2013.05.05

**Published:** 2013-05-20

**Authors:** 靖颖 农, 娜 秦, 敬慧 王, 新杰 杨, 卉 张, 羽华 吴, 嘉林 吕, 权 张, 树才 张

**Affiliations:** 101149 北京，首都医科大学附属北京胸科医院/北京市结核病胸部肿瘤研究所肿瘤内科 Department of Medical Oncology, Beijing Chest Hospital, Capital Medical University/Beijing Tuberculosis and Thoracic Tumor Research Institute, Beijing 101149, China

**Keywords:** 盐酸埃克替尼, 肺肿瘤, 疗效, Icotinib, Lung neoplasms, Efficacy

## Abstract

**背景与目的:**

盐酸埃克替尼是第三个在临床上应用于晚期非小细胞肺癌（non-small cell lung cancer, NSCLC）治疗的单靶点表皮生长因子受体酪氨酸激酶抑制剂（epidermal growth factor receptor tyrosine kinase inhibitor, EGFR-TKI），关于其在复发肺癌患者中的疗效及生存的临床研究报道仍甚少。本研究回顾性分析盐酸埃克替尼在晚期复发NSCLC中的疗效及安全性，并探讨影响因素。

**方法:**

对2009年3月-2012年7月北京胸科医院收治的60例接受盐酸埃克替尼治疗的晚期复发NSCLC患者的临床资料进行回顾性分析。

**结果:**

60例均可评价疗效，总有效率45%，疾病控制率80%，中位无进展生存期（progression free survival, PFS）6.7个月。女性患者的有效率、PFS优于男性（*P*值分别为0.014、0.013）；二线治疗组的有效率和疾病控制率优于三线及以上组（*P*值分别为0.020、0.024）；年龄≥65岁与 < 65岁，PS评分 < 2分与≥2分，埃克替尼二线治疗与三线以上治疗组患者的疗效及PFS无明显差别。*EGFR*敏感突变患者的有效率、疾病控制率及无进展生存均明显优于*EGFR*野生型患者（*P*值分别为0.006、 < 0.001、0.002）；外显子19缺失突变组与外显子21 L858R突变组近期疗效无明显差别，而前者PFS明显更长（*P*=0.020）。*EGFR*敏感突变、外显子19缺失突变是具明显生存优势的独立因子（*P*值分别为0.009、0.012）。毒副反应主要为轻度皮疹及腹泻。

**结论:**

盐酸埃克替尼是治疗晚期复治NSCLC的有效药物，安全性好，尤其在携带敏感突变的患者中疗效更佳。

## Abstract (en)

**Background and objective:**

Icotinib hydrochloride is the third single target EGFR-TKI used in clinical treatment of advanced non-small cell lung cancer (NSCLC). Clinical research reports on its efficacy and survival in patients with Recurrent Advanced NSCLC are still little.The aim of this study is to evaluate the efficacy and survival of Icotinib hydrochloride for patients with advanced non-small cell lung cancer who failed to previous chemotherapy and explore the association of clinical features with the efficacy and survival.

**Methods:**

The clinical data of 60 NSCLC patients referred to the Beijing Chest Hospital, Capital Medical University from March 2009 to July 2012 were retrospectively analyzed.

**Results:**

The overall response rate (ORR) was 45.0% and the disease control rate (DCR) was 80.0%. The median progression-free survival (PFS) time was 6.7 months. RR and PFS in female were superior to male (*P*=0.014, 0.013, respectively). RR, DCR in 2^nd^-line subgroup were superior to ≥3^rd^-line subgroup (*P*=0.020, 0.024, respectively). RR, DCR and PFS in *EGFR* mutation carriers were significantly superior to wild-type patients (*P*=0.006, < 0.001, 0.002, respectively). There was no statistical difference in RR and PFS between those age < 65 and ≥65 or PS < 2 and PS≥2. There was no statistical difference in RR and DCR between exon 19 deletion and exon 21 mutations, while the former had much longer PFS (*P*=0.020). *EGFR* mutation and exon 19 deletion are the independent prognostic factors to significantly improve the PFS (*P*=0.009, 0.012, respectively). The side effects were generally mild and consisted of rash and diarrhea.

**Conclusion:**

Icotinib hydrochloride is effective especially in *EGFR* mutation carriers and well tolerated in patients with recurrent advanced non-small-cell lung cancer.

近些年来，表皮生长因子受体酪氨酸激酶抑制剂（epidermal growth factor receptor tyrosine kinase inhibitor, EGFR-TKI）已广泛应用于晚期非小细胞肺癌（non-small cell lung cancer, NSCLC）的二线治疗，延长了晚期患者的生存时间，与标准二线化疗相比具有更好的耐受性，在亚裔、女性、腺癌、不吸烟患者中的的疗效更优^[1-3]^。盐酸埃克替尼（商品名为凯美纳）作为继吉非替尼、厄洛替尼后第三个在临床应用于晚期NSCLC治疗的单靶点EGFR-TKI，是我国第一个自主知识产权的小分子靶向抗癌新药。现对北京胸科医院2009年3月-2012年7月来收治的接受盐酸埃克替尼治疗，临床资料完整的晚期复治NSCLC患者的疗效及安全性进行回顾性分析，并探讨其疗效影响因素。

## 1 资料与方法

### 1.1 纳入标准

① 经组织学或细胞学证实的Ⅲb期或Ⅳ期NSCLC（根据IASLC第7版非小细胞肺癌TNM临床分期标准）；②二线及以上使用盐酸埃克替尼，单药口服埃克替尼125 mg，tid，30天以上；③至少具有一个可评价病灶。

### 1.2 排除标准

① 无病理诊断；②临床资料不完整；③无可评价病灶；④重复应用TKI治疗的患者。

### 1.3 临床资料

2009年3月-2012年7月，共60例患者符合以上入排标准。临床资料包括患者性别、年龄、病理类型、肿瘤分期、吸烟史、ECOG PS评分、既往化疗史等。所有患者在治疗前完成血常规、肝肾功能、胸部CT、腹部彩超、头部CT或MRI及骨扫描检查，评估PS评分。用药期间定期复查上述指标，服药1个月后进行影像学检查，随后每2月复查一次，直至出现疾病进展或不可耐受的毒副反应。

### 1.4 疗效评价

按照实体瘤治疗疗效评价（RECIST 1.1）标准评价近期疗效，分为完全缓解（complete response, CR）、部分缓解（parital response, PR）、疾病稳定（stable disease, SD）和疾病进展（progressive disease, PD）。客观缓解率（objective response rate, ORR）包括CR和PR，疾病控制率（disease control rate, DCR）包括CR、PR和SD。无进展生存期（progression free survival, PFS）为从首次服药到客观证据证实疾病进展或任何原因引起死亡的时间，到截止时间时疾病未进展/死亡的患者以最后一次肿瘤评估的日期计算PFS。与药物相关的毒性反应根据美国国立肿瘤研究院毒性分级标准（CTC）3.0进行分级。

### 1.5 统计学方法

使用SPSS 16.0进行统计学处理。各临床特征与TKI疗效相关性分析采用卡方检验或*Fisher*精确检验；生存分析采用*Kaplan-Meier*法，*Log-rank*检验组间差异，*Cox*回归进行生存的多因素分析。以*P* < 0.05为差异有统计学意义。

## 2 结果

### 2.1 临床特征

60例患者中，中位年龄58岁（36岁-86岁），女性34例（56.7%），Ⅳ期56例（93.3%），二线治疗37例（60.0%），PS评分0分-1分34例（56.7%），不吸烟者34例（56.7%），腺癌54例（90.0%）。60例患者中，有32例（53.3%）患者知情同意进行*EGFR*突变检测，23例存在敏感突变，9例为野生型；外显子19缺失突变14例，外显子21 L858R突变9例（表 1）。

### 2.2 疗效

全部患者均可评价疗效：全组无CR病例，PR 27例（45.0%），SD 21例（35.0%），PD 12例（20.0%）。ORR 45.0%，DCR 80.0%（表 1）。卡方检验显示，女性患者有效率高于男性（*P*=0.014），二线组的ORR及DCR均优于三线及以上组（*P*值分别为0.020、0.024）；年龄≥65岁与 < 65岁患者，PS评分 < 2分与≥2分，吸烟与不吸烟、腺癌与鳞癌患者的RR、DCR差异无统计学意义。23例突变患者和9例野生型患者的ORR分别为65.2%和11.1%（*P*=0.006），DCR分别为100%和44.4%（*P* < 0.001）（表 2）。

**1:** 60例盐酸埃克替尼治疗的晚期复治NSCLC患者特征 The characteristics of 60 patients with recurrent advanced NSCLC treated with Icotinib hydrochloride

Variable	Group	*n*=60（%）	*EGFR* mutation status		
			Mut (+)（*n*=23）	Wild-type (*n*=9)	Unknown (*n*=28）
Gender	Male	26（43.3）	6	6	14
	Female	34（56.7）	17	3	14
Age	＜65 yr	42（70.0）	16	7	19
	≥65 yr	18（30.0）	7	2	9
PS score	0-1	34（56.7）	15	5	14
	2-3	26（43.3）	8	4	14
Smoke	Yes	26（43.3）	9	5	12
	No	34（56.7）	14	4	16
Pathological type	Adeno	54（90.0）	21	8	25
	Squamous	6（10.0）	2	1	3
Time of treatment	2^nd^-line	37（61.7）	19	2	16
	> 2^nd^-line	23（38.3）	4	7	12
NSCLC: non-small cell lung cancer; EGFR: epidermal growth factor receptor.					

**2:** 60例埃克替尼治疗晚期复治NSCLC患者疗效及PFS分析 Analysis of efficacy and PFS for 60 patients with recurrent advanced NSCLC treated with Icotinib hydrochloride

Variable		ORR (%)	*P*	DCR (%)	*P*	PFS (mo)	Range (mo)	*P*
Gender	Male	26.9	0.014	73.1	0.241	3.5	1.0-18.8	0.013
	Female	58.8		85.3		10.8	1.0-23.5	
Age	< 65 yr	40.5	0.282	76.2	0.260	5.3	1.0-23.5	0.769
	≥65 yr	55.6		88.9		7.1	1.0-13.3	
PS score	0-1	50.0	0.373	85.3	0.241	5.3	1.0-23.5	0.745
	≥2	38.5		73.1		7.3	1.0-15.3	
Smoke	Yes	34.6	0.157	80.8	0.896	5.3	1.0-18.8	0.410
	No	52.9		79.4		7.1	1.0-23.5	
Pathological type	Adeno	46.3	0.545	79.6	0.830	7.3	1.0-23.5	0.722
	Squamous	33.3		83.3		3.0	1.4-18.8	
Time of treatment	2^nd^-line	55.6	0.020	88.9	0.024	7.5	1.0-18.8	0.297
	> 2^nd^-line	26.1		65.2		3.5	1.0-23.5	
*EGFR* mutation status	Mut (+)	65.2	0.006	100.0	＜0.001	10.8	2.3-23.5	0.002
	Wild-type	11.1		44.4		1.4	1.0-9.8	
	Unknown	39.3		75.0		7.3	1.0-18.8	
ORR: objective response rate; DCR: disease control rate; PFS: progression free survival.								

全部患者随访至2012年12月30日，45例（75.0%）患者出现疾病进展或死亡，其余15例患者仍在服药治疗。中位PFS 6.7个月（1个-23.5个月）。*Log-rank*检验显示女性较男性的PFS更长（*P*=0.013）。而年龄≥65岁与 < 65岁患者，PS评分 < 2分与≥2分，吸烟与不吸烟，腺癌与鳞癌，二线与三线及以上组间PFS无统计学差异（表 2）。*Kaplan-Meier*曲线见图 1-图 3。

**1:**
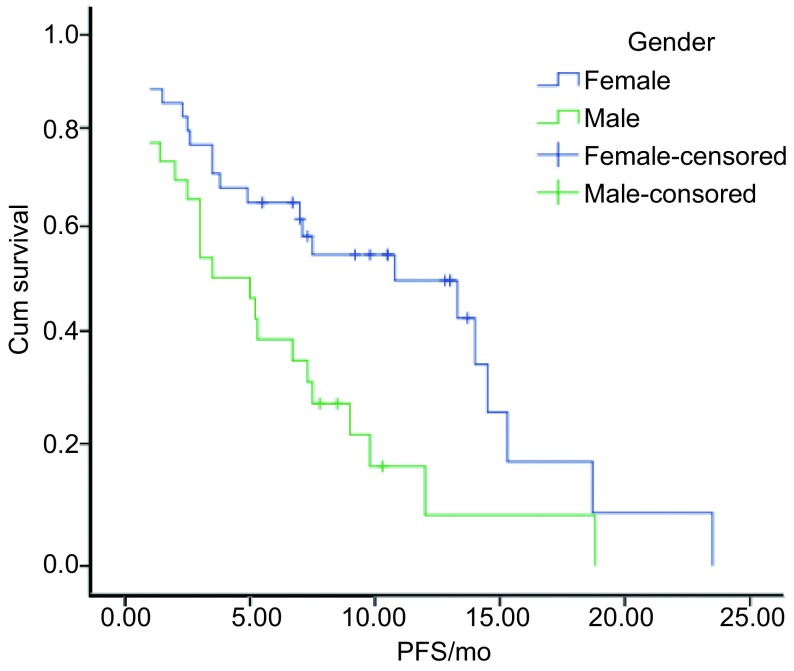
不同性别的*Kaplan*-*Meier*生存曲线 *Kaplan*-*Meier* survival curves of different gender

**2:**
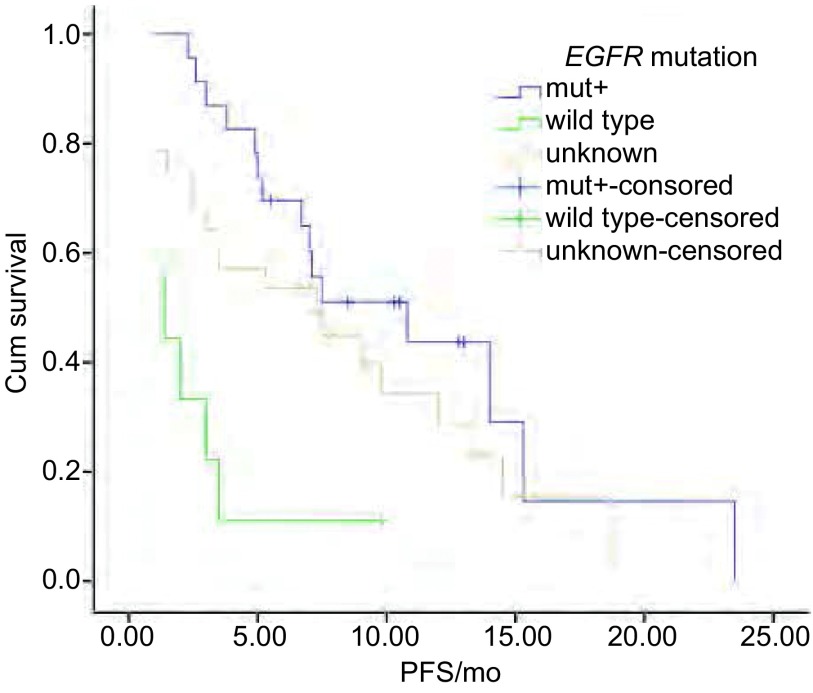
不同突变状态的*Kaplan*-*Meier*生存曲线 *Kaplan*-*Meier* survival curves of different *EGFR* mutation status

**3:**
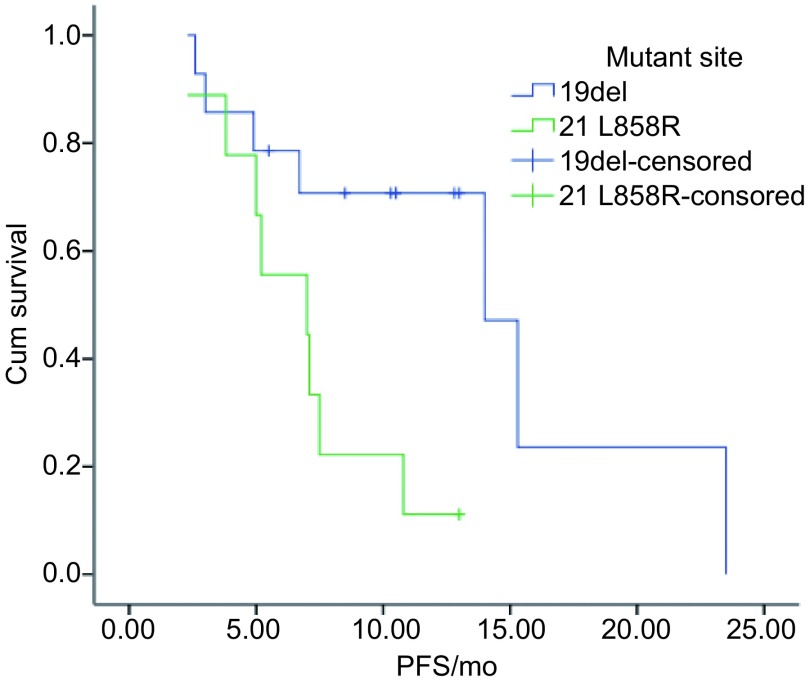
不同突变位点的*Kaplan*-*Meier*生存曲线 *Kaplan*-*Meier* survival curves of different mutant sites

随访至2012年12月30日，32例接受EGFR检测患者中，共有23例患者出现疾病进展。23例突变患者中有15例出现疾病进展。突变患者与野生型患者的PFS分别为10.8个月和1.4个月；外显子19缺失突变与外显子21点突变患者PFS分别为14.0个月和7.0个月。*Log-rank*结果显示*EGFR*突变型较野生型（*P*=0.002）、外显子19缺失突变较外显子21 L858R突变者的PFS明显更长（*P*=0.002），*Cox*多因素分析显示EGFR敏感突变者（*P*=0.009）和外显子19缺失突变者（*P*=0.012）是具明显生存优势的独立因素。

### 2.3 毒副反应

本文观察到的埃克替尼相关副反应发生率为50%（30例），主要为皮疹（38.3%）、腹泻（14.2%）、皮肤瘙痒（2.8%）、转氨酶异常（1.7%），均为1级-2级，对症处理均可减轻。无一例因副反应减停药物。

## 3 讨论

目前报道的晚期NSCLC二线化疗有效率仅约10%，EGFR-TKIs的临床应用给一线化疗失败的晚期NSCLC患者带来了更好的疗效，延长了生存期。盐酸埃克替尼是作用于表皮生长因子受体酪氨酸激酶的小分子抑制剂，其分子结构式类似于吉非替尼和厄洛替尼^[4]^。ICOGEN研究是对比盐酸埃克替尼和吉非替尼二、三线治疗NSCLC患者的非劣效性设计的Ⅲ期临床试验，其结果显示二者疗效相当，埃克替尼的安全性更佳^[5]^。近期有临床观察显示埃克替尼在晚期NSCLC中具良好的临床疗效与安全性^[6]^。

近期疗效上，ICOGEN埃克替尼治疗组有效率27.6%，疾病控制率75.4%。本研究中全组疾病控制率80%，与ICOGEN相似，而有效率则更高（45%），可能与突变人数较多（23例）有关，而突变状态未知的28例中，腺癌所占比例偏高（89.3%），也可能对结果产生影响。亚组分析中，女性有效率高于男性，与多数研究类似，可能与女性*EGFR*突变率更高有关。二线组的有效率及控制率优于三线及以上组，分析发现前者有突变者比例高达51.4%（19/37），后者仅为17.4%（4/23），两组数据不均衡。鳞癌亚组有效率达33.3%（2/6），原因是6例鳞癌患者中有2例突变，该2例有效，且鳞癌例数少，导致鳞癌组有效率与腺癌组无统计学差异。日本一项研究^[7]^显示有突变的鳞癌接受EGFR-TKI治疗缓解率30%，控制率70%，本研究与此相似。本组患者中，吸烟与不吸烟患者的疗效无差异，可能与两组突变患者所占比例相近有关。

全组患者PFS达到6.7个月，略高于ICOGEN中埃克替尼治疗组（154天），可能与突变比例较高有关。其中鳞癌组PFS 3.0个月，与Shukuya等的研究^[7]^相一致。女性PFS明显好于男性，分析发现女性组中*EGFR*突变者所占比例（17/34）明显高于男性组（6/26），可能对生存结果产生影响。

对于老年、PS评分差的患者，因化疗耐受性及依从性进一步下降，二线治疗选择更加困难。多项报道^[8, 9]^显示老年二线以上接受EGFR-TKI治疗同样有较好的疗效和生存。IPASS研究^[10]^发现*EGFR*突变率在≥65岁患者为68.5%， < 65岁患者56.7%，老年优势人群的*EGFR*突变几率可能更高。本组中有≥65岁患者共18例，有效11例（55.6%），中位PFS 7.1个月。该组中EGFR敏感突变7例，野生型2例，EGFR状态未知9例。老年人接受低毒性的TKI治疗有效率不低于甚至可好于相对年轻患者。在BR.21研究中^[11]^，PS 0分-1分与2分-3分组的有效率无差异，本研究与之相似。其中PS≥2分患者的有效率仍可达38.5%，PFS达7.3个月。实践中这些患者往往因PS评分差失去化疗机会，TKI给他们带来了很好的疗效，生存也得到延长。

*EGFR*突变是预测TKI在NSCLC中疗效最有力的证据。Lynch等^[12]^首先揭示了*EGFR*基因突变与疗效之间的关系。本文将全组分为有*EGFR*突变、野生型和EGFR未知型进行分析，发现无论是近期疗效还是PFS，*EGFR*突变组与野生型组均有明显差别。尽管证实*EGFR*基因突变与疗效关系的研究多在一线治疗中, 但一些研究的亚组分析在*EGFR*基因突变与二、三线TKI治疗的关系上仍给出了提示。BR.21研究^[11]^中敏感突变亚组的有效率37.5%，INTEREST研究^[1]^亚组分析显示，二三线使用吉非替尼治疗的*EGFR*基因敏感性突变患者PFS更长。ICOGEN研究中*EGFR*敏感突变者ORR为59.1%，而野生型仅5.1%。本研究中EGFR敏感突变者共23例，有效率65.2%，与ICOGEN结果相似，也接近有突变的一线患者的结果。中位PFS达到10.8个月，提示二、三线接受埃克替尼治疗的*EGFR*敏感突变患者可获得很好的疗效。突变组中，有14例外显子19缺失突变，另9例外显子21 L858R点突变，这两个亚组有效率相当（分别为64.3%，66.7%）。而外显子19缺失突变者PFS 14.0个月，明显长于外显子21 L858R点突变的7.0个月（*P*=0.02），Jackman等^[13]^报道在接受TKI治疗的*EGFR*突变患者中，外显子19缺失患者较L858R突变者的生存期延长，本研究结果与之相似。

本研究中有9例为*EGFR*野生型患者，中位PFS仅为1.4个月。其中1例有效（11.1%），不能排除该病例肿瘤异质性明显或检测方法假阴性可能。2012 ASCO报道TAILOR研究中，*EGFR*野生型患者二线接受TKI治疗与多西他赛单药相比有效率低（仅为2.2%）^[14]^。*EGFR*野生型患者应该探讨其它的治疗策略。本研究中还有28例*EGFR*突变状态未知，11例（39.3%）有效，21例（75%）疾病控制，PFS 7.3个月。分析发现优势人群（女性、腺癌、非吸烟者）所占比例偏高。对于有优势临床特征的患者，二线治疗优先考虑EGFR-TKI（如盐酸埃克替尼）治疗可能会得到比化疗更佳的疗效和生存。

本文观察到的盐酸埃克替尼副反应轻微，相关毒副作用的发生率为50%（30例），主要为皮疹（38.3%）、腹泻（14.2%），无1例因副反应减停药物。ICOGEN试验报告的盐酸埃克替尼药物相关毒副反应发生率为60.5%，皮疹39.5%，腹泻18.5%，本组的毒副反应不高于ICOGEN试验。

综上所述，本项回顾性分析显示盐酸埃克替尼在晚期复治的NSCLC患者中具有较好的疗效，副反应轻，安全性好，*EGFR*突变者获益更为明显，老年人及PS评分较差的患者也能得到较好疗效，是晚期复治NSCLC的较好治疗选择。随着临床上的广泛应用，埃克替尼的疗效及安全性将得到进一步的验证。
